# Effects of Epigallocatechin Gallate Against Lung Cancer: Mechanisms of Action and Therapeutic Potential

**DOI:** 10.3390/nu18030378

**Published:** 2026-01-23

**Authors:** Dordaneh Mirbabaei Ghafghazi, Newman Siu Kwan Sze, Evangelia Tsiani

**Affiliations:** 1 Department of Health Sciences, Brock University, St. Catharines, ON L2S 3A1, Canada; dm19xq@brocku.ca (D.M.G.);; 2 Centre for Bone and Muscle Health, Brock University, St. Catharines, ON L2S 3A1, Canada

**Keywords:** lung cancer, epigallocatechin-3-gallate, proliferation, apoptosis, migration signaling cascades

## Abstract

Epigallocatechin-3-gallate (EGCG), the major bioactive polyphenol in green tea, has garnered significant attention for its potential anticancer properties. This review summarizes the current evidence from in vitro, in vivo, and clinical trials examining the effects of EGCG on lung cancer. EGCG exerts its anticancer effects through various mechanisms, including the inhibition of cell proliferation, induction of apoptosis, suppression of metastasis, and modulation of signalling pathways such as epidermal growth factor receptor (EGFR), phosphoinositide 3-kinase/protein kinase B (PI3K/Akt), mitogen-activated protein kinase (MAPK), and nuclear factor kappa B (NF-κB). Additionally, EGCG has been shown to enhance the efficacy of conventional chemotherapeutic agents and mitigate drug resistance. However, challenges related to its bioavailability and metabolic stability remain. Ultimately, this review aims to provide a comprehensive overview of the effects of EGCG against lung cancer.

## 1. Introduction

Despite major advances in targeted therapies and immunotherapies [1,2], lung cancer is still the leading cause of cancer mortality globally and accounts for 18.4% of total cancer-related deaths [2]. Consequently, there is growing interest in alternative strategies for cancer prevention and treatment, including the potential use of natural compounds such as epigallocatechin gallate (EGCG) as alternative or complementary approaches. The two main subtypes of lung cancer non-small cell lung carcinoma (NSCLC), and small-cell lung carcinoma (SCLC), account for 85% and 15% of the total cases of lung cancer, respectively [2]. NSCLC is subdivided into adenocarcinoma, squamous cell carcinoma, and large cell carcinoma [2], which make up 40%, 25–30%, and 10–15% of the cases, respectively [3]. Each subtype has unique features which can be used in its diagnosis and treatment [4]. The risk of developing lung cancer is strongly associated with the duration and the amount an individual smokes, and it remains elevated even long after quitting [4]. Moreover, exposure to second-hand smoke significantly increases the likelihood of development of lung cancer in individuals who do not smoke [4]. Other risk factors for developing lung cancer include occupational exposure to substances such as asbestos, chromium, arsenic, and diesel exhaust [4]. The second most common cause of lung cancer is radon gas, a radioactive gas present in soil and construction materials [4]. Genetic factors can also significantly increase the risk of developing lung cancer [4]. For instance, genetic mutations in the *p53* tumour suppressor gene, epidermal growth factor receptor (*EGFR*), Kirsten rat sarcoma virus (*KRAS*), and anaplastic lymphoma kinase (*ALK*) rearrangements are all associated with its development [4]. Frequent mutations are those found in EGFR, *ALK*, c-ros oncogene 1 (*ROS1*), and *KRAS*, which are responsible for promoting unregulated cell growth and metastasis [4]. Ultimately, NSCLC develops as a result of a combination of environmental, genetic, and cellular influences [4]. Treatments for lung cancer consist of chemotherapy, targeted therapy, chemoradiotherapy, immunotherapy, anti-angiogenic therapy, and combination therapy [1].

**Figure 1:**
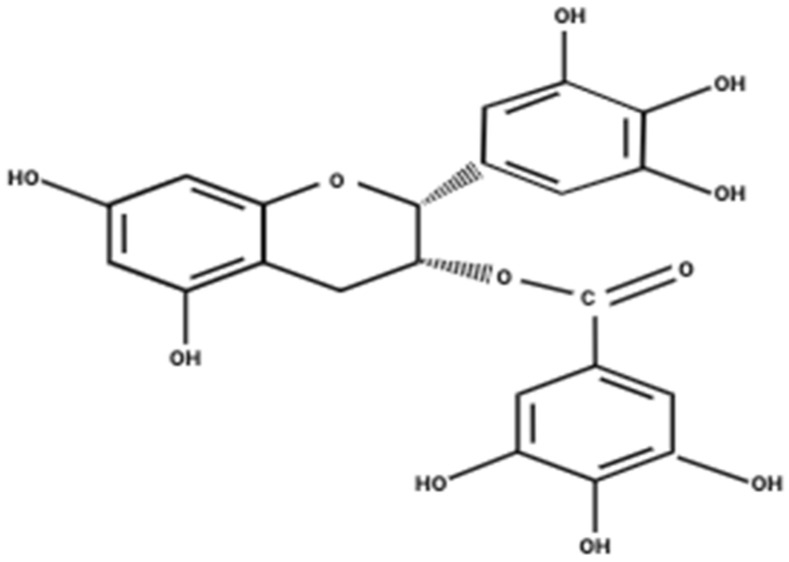
Chemical structure of epigallocatechin gallate (EGCG). Created in BioRender. Dordaneh Mirbabaei Ghafghazi. (2026) https://www.biorender.com.

Polyphenols are naturally occurring compounds produced by plants, shown to be effective in regulating oxidative and inflammatory stress [5]. Studies have revealed an inverse association between the consumption of polyphenols and the risk of developing chronic diseases such as diabetes, cardiovascular disease, and various cancers [5]. From a structural perspective, polyphenols contain at least one phenyl ring and one or more hydroxyl groups [5]. Polyphenols are classified into phenolic acids, lignans, stilbenes, and flavonoids [6]. (−)-epigallocatechin-3-gallate (EGCG) is the major polyphenol found in green tea (*Camellia sinensis*) and most of the potential health benefits of green tea are attributed to it [7]. EGCG comprises three aromatic rings which are linked together by a pyran ring (Figure 1) [8]. This polyphenol has been shown to possess antioxidant, anti-inflammatory, and anticancer properties [9]. A systematic literature search was conducted using PubMed with the key words “EGCG” and “Lung Cancer,” including only peer-reviewed English-language studies and excluding reports not directly relevant to the topic. Evidence from in vitro, in vivo, and available clinical studies was integrated to provide a comprehensive and critical assessment of the effects of EGCG in lung cancer.

This review summarizes recent findings on the effects of EGCG in lung cancer, particularly its mechanistic actions targeting key biological processes involved in cancer. EGCG inhibits lung cancer through multiple pathways, including suppression of cell proliferation and cell-cycle progression, induction of apoptosis and ferroptosis, inhibition of migration, invasion and EMT, anti-angiogenic activity, targeting of cancer stem cell properties, modulation of oncogenic signalling pathway, regulation of cellular metabolism and oxidative stress, epigenetic modifications and non-coding RNA activity, immune modulation (e.g., PD-L1 expression), and enhancement of chemosensitivity and overcoming drug resistance. Examining the mechanisms of EGCG action against lung cancer may provide new insights and directions for leveraging its effects in lung cancer prevention and therapy. Although several reviews published over the past decade have summarized the anticancer properties of EGCG in lung cancer, the present review extends beyond a descriptive overview by providing a critical and comparative synthesis across lung cancer subtypes, experimental models, and molecular contexts. Specifically, it distinguishes between EGCG effects that are consistently reproducible across multiple models and those that are highly dependent on cell line, genetic background, dosage, or treatment context, while reassessing contradictory findings reported in the literature. This review further integrates recent evidence published between 2020 and 2024 and places greater emphasis on translational considerations, including pharmacokinetic limitations, bioavailability challenges, and emerging strategies such as nanoformulations, molecular conjugates, and combination therapies aimed at improving clinical applicability. By integrating mechanistic insights with realistic therapeutic constraints, this work provides an updated, clinically grounded perspective that highlights the requirement of future human research.

## 2. Effects of Epigallocatechin Gallate Against Lung Cancer

### 2.1. Proliferation and Cell-Cycle Control

Uncontrolled proliferation is a hallmark of cancer. Numerous studies have shown that EGCG can inhibit the growth of lung cancer cells by inducing cell-cycle arrest in a dose-dependent manner. EGCG suppresses the viability of both NSCLC and SCLC cell lines, with reported IC50 values of approximately 22 µM in more sensitive models such as H661 and H1299 (Table 1) [10,11,12]. In contrast, reduced responsiveness is observed in other cell lines such as H441, highlighting cell line-dependent variability in antiproliferative efficacy [10,11,12]. Okabe et al. demonstrated that EGCG and related catechins inhibited lung adenocarcinoma PC-9 cell growth by arresting the cells in G2/M phase, indicating a block at the G2/M checkpoint [10]. Consistently, EGCG has been observed to arrest lung cancer cells at various checkpoint points: G2/M phase accumulation, S-phase or G0/G1 arrest in various models. In SCLC H69 cells, EGCG induced a dose-dependent cytotoxicity accompanied by S-phase arrest and reduced telomerase activity. EGCG exposure can impair replicative immortality in cancer cells [13]. In EGCG-treated NSCLC cells, G0/G1 phase arrest is also reported. An amount of 40 µM EGCG can cause a significant G0/G1 accumulation [14]. Long-term EGCG exposure suppresses colony formation and anchorage-dependent growth of lung cancer cells, as seen in both EGFR-wild-type and mutant NSCLC cell lines [15,16]. Importantly, in vivo xenograft studies confirm that EGCG can slow tumour growth. EGCG dietary intake or intraperitoneal administration have been shown to significant induce tumour growth inhibition in lung cancer xenografts mice [17]. Tumours treated with EGCG reduced Ki-67 and proliferated cell nuclear antigen (PCNA) staining, reflecting the cell-cycle arrest and antiproliferative effect [18].

Mechanistically, EGCG downregulated cyclins and other cell-cycle regulators to arrest cells at the cell-cycle checkpoints. EGCG alone or in combination with other compounds can reduce the expression of Cyclin D1 and Cyclin B1, which drive G1/S and G2/M transitions. The combination of EGCG with curcumin markedly decreased CyclinD1 and B1 levels in A549 and H460 NSCLC cells, correlating with cell-cycle arrest at both G1 and S/G2 phases and a reduction in DNA synthesis rates [19]. Prolonged EGCG treatment was also found to diminish Cyclin D1 expression in EGFR-dependent lung cancer cells, in conjunction with loss of activated EGFR signalling (a pathway that normally upregulates Cyclin D1) [16]. These molecular changes blocked cell-cycle progression, inhibiting cancer cell proliferation. EGCG has also been shown to increase expression of cell-cycle inhibitors like p21^Cip1/Waf1^, especially when used in combination treatments [20], contributing to growth arrest. EGCG also induced cell-cycle arrest in therapy-resistant cells. EGCG inhibited the proliferation of cisplatin-resistant NSCLC cancers in a concentration-dependent manner, reducing their growth to levels similar to parental cells [21,22]. Moreover, co-treatment of resistant cells with EGCG and other chemotherapy drugs induced cell-cycle arrest more effectively than monotherapy [21]. By inhibiting cell-cycle progression at checkpoints and suppressing the expression of cyclins and proliferative markers, EGCG suppresses lung cancer cell proliferation. Across NSCLC and SCLC models, lung cancer cell proliferation is consistently suppressed by EGCG through disruption of cell-cycle progression, reduced colony formation, and decreased Ki-67 and PCNA expression in vitro and in vivo [10,11,12,13,14,15,16,17,18]. However, both antiproliferative sensitivity and the specific cell-cycle checkpoint affected (G0/G1, S, or G2/M) vary by cell line. Greater responsiveness is observed in H661 and H1299 cells (IC_50_~22 µM) compared with less sensitive models such as H441, underscoring model-dependent variability that limits direct cross-study comparison and clinical extrapolation [10,11,12].

### 2.2. Apoptosis and Ferroptosis

Induction of regulated cell death is another major mechanism by which EGCG inhibits lung cancer progression. Several studies have demonstrated that EGCG activates both apoptotic and ferroptotic pathways in NSCLC and SCLC cell, thereby suppressing cell survival and promoting cancer cell elimination. EGCG increases apoptotic rates in NSCLC models in a dose-dependent manner, as seen in H661 cells where apoptosis rose from 23% at 30 µM to 82% at 100 µM (Table 1) [11]. Consistently, EGCG triggered intracellular and mitochondrial ROS generation in H1299 cells, and antioxidant treatment blocked apoptosis, indicating that ROS production is required for EGCG-induced apoptotic signalling [17].

Mechanistically, EGCG promotes apoptotic signalling by increasing cleaved caspase-3 and Bcl-2 associated X protein (Bax), while decreasing B-cell lymphoma-extra large (Bcl-xL) [23] and increasing B-cell lymphoma 2 (Bcl-2) [24]. Treatment of A549 cells with EGCG also led to decreased viability, induced apoptosis, and suppressed Bcl-xL mRNA [25]. EGCG also enhances markers of apoptosis when used in combination regimens. For instance, co-treatment with celecoxib increased growth arrest and DNA damage-inducible 153 (GADD153) expression and apoptotic cell death in PC-9 cells [26]. EGCG with luteolin similarly increased death receptor 5 (DR5) activation, and cytochrome c release in NSCLC cell lines, strengthening the apoptotic response [27]. EGCG-mediated apoptosis is further enhanced when combined with ascorbic acid. In SPC-A-1 cells, EGCG alone or in combination with ascorbic acid increased caspase-9 activity and promoted apoptotic signalling activation of the c-Jun *N*-terminal kinase (JNK), P38 and extracellular signal-regulated kinase (ERK) pathways, indicating that multiple mitogen-activated protein kinase (MAPK)-mediated cascades contribute to this effect [28]. Similarly, EGCG combined with Leptomycin B significantly reduced A549 cell viability, increased ROS levels, decreased cytochrome P450 3A4 (*CYP3A4*), superoxide dismutase (*SOD*), and glutathione peroxidase 1 (*GPX1*) mRNA, and enhanced p21 expression compared with Leptomycin B alone [29].

EGCG-induced apoptosis is also mediated through p53-dependent mechanisms. In A549 cells, EGCG suppresses cell viability and selectively induces caspase-3 and caspase-7 activity compared with other green tea catechins, indicating that apoptosis is specifically triggered by EGCG [30]. EGCG treatment upregulates *p53* expression, and RNAi-mediated knockdown of *p53* abolishes EGCG-induced caspase activation, demonstrating that *p53* plays a critical role in mediating EGCG-induced apoptotic signalling in this model [30]. Similarly, in H1650, A549, and H460 cells, EGCG enhances p53 nuclear localization and stability, while reducing nuclear accumulation of mouse double minute 2 (MDM2), and enhancing phosphorylation of p53 at Ser15 and Ser20 [31]. EGCG also disrupts the interaction between p53 and MDM2, suppressing MDM2-mediated ubiquitination and increasing p53 transcriptional activity [31].

EGCG also enhances apoptosis in therapy-resistant and cancer stem-like cells. In cisplatin-resistant A549 cells, EGCG combined with cisplatin increased apoptosis [22]. Additional findings show that EGCG enhances apoptosis in resistant cells through regulation of copper transporter 1 (CTR1) and *hsa-miR-98-5p* [32]. In lung CSCs, EGCG reduced stem cell markers and increased Bax, Caspase-8, cleaved Caspase-9, and cleaved Caspase-3 [33]. Combination treatments further strengthened EGCG-dependent apoptosis: co-treatment with Am80 upregulated GADD153, DR5, and p21^waf1^ expression [20], while metformin enhanced EGCG-induced ROS, caspase-3 activation, and poly(ADP-ribose) polymerase 1 (PARP1) cleavage [18].

Enhanced EGCG formulations potentiate apoptosis. Nanoformulated EGCG displayed greater efficacy in reducing A549 cell viability compared with free EGCG, accompanied by increased intracellular ROS [34]. Both nano and free EGCG upregulated nuclear factor erythroid 2-related factor 2 (Nrf2) and heme oxygenase-1 (HO-1), while decreasing Kelch-like ECH-associated protein 1 (Keap) expression, and induced DNA damage [34]. Notably, nano EGCG led to higher expression of pro-apoptotic proteins Bax, Bcl-2 homologous antagonist/killer (Bak), Bcl-2-interacting mediator of cell death (Bim), and p53 upregulated modulator of apoptosis (Puma), highlighting the enhanced apoptotic potential of this delivery method [34].

In addition to apoptosis, EGCG induces ferroptosis in lung cancer cells. EGCG downregulated glutathione peroxidase 4 (GPX4) and solute carrier family 7 member 11 (SLC7A11), increased acyl-CoA synthetase long-chain family member 4 (ACSL4), and elevated intracellular iron, ROS, and malondialdehyde in A549 and H129 cells, all consistent with ferroptotic cell death [48]. Supporting this mechanism, EGCG inhibited leptin-induced cell survival, colony formation, migration, and invasion through targeting signal transducer and activator of transcription 1 (STAT1) and *SLC7A11*, indicating ferroptosis involvement in NSCLC progression [49]. Collectively these findings demonstrate that EGCG suppresses lung cancer growth by inducing apoptosis and ferroptosis across diverse cell models (Table 1).

Consistent induction of apoptosis across NSCLC and SCLC models is observed following EGCG treatment, with reproducible involvement of mitochondrial ROS generation, caspase activation, and p53-dependent signalling [11,17,23,24,25,28,30,31]. These effects are strengthened in therapy-resistant and stem-like cells and by combination treatments or nanoformulations, indicating enhanced efficacy under cellular stress conditions [18,20,22,32,33,34]. In contrast, ferroptosis has been reported in fewer models and appears highly context-dependent, influenced by iron metabolism and antioxidant capacity [48,49].

### 2.3. Cell Migration, Invasion, and EMT (Metastasis)

Metastasis is a defining feature of lung cancer progression, and numerous studies have shown that EGCG can inhibit migration, invasion, and epithelial to mesenchymal transition (EMT). In highly metastatic 95-D cells, EGCG decreases matrix metalloproteinase 9 (*MMP-9*) mRNA and protein expression, and significantly suppresses invasion, an effect accompanied by reduced intracellular oxidants (Table 1) [50]. Consistently, reduction in invasive potential have been reported in CL1-5 cells, where EGCG downregulates MMP-2 expression at the transcriptional and protein levels [51]. EGCG also interferes with EMT, a key driver of metastasis. In A549 and H1299 cells, EGCG prevents transforming growth factor β (TGF-β)-induced EMT, restoring E-cadherin expression and suppressing markers such as vimentin and EMT-associated transcription factors including Snail, Slug, zinc finger E-box binding homeobox 1 (Zeb1), and Twist1 [52]. EGCG further inhibits TGF-β1-induced acetylation of Smad2/3 through suppression of p300 and CREB-binding protein (CBP), thereby blocking downstream EMT transcriptional reprogramming [53]. Additional EMT-promoting stimuli are similarly counteracted: EGCG inhibits nicotine-induced EMT in A549 cells, decreasing hypoxia-inducible factor 1 α (HIF-1α), vascular endothelial growth factor (VEGF), cyclooxygenase-2 (COX-2), vimentin, and restoring the expression of β-catenin, and p53 [54]. Beyond effects on EMT markers and transcriptional factors, EGCG alters physical and structural properties that enable cell migration. In H1299 and Lu99 cells, EGCG increases cellular stiffness and decreases vimentin and Slug at the leading edge of migrating cells, indicating reduced cytoskeletal flexibility and impaired motility [55]. EGCG also suppresses hepatoma-derived growth factor (HDGF) expression, leading to reduced migration of A549 cells [35]. Enhanced formulations further strengthen these anti-metastatic effects: nano-EGCG more effectively inhibits migration and invasion of H1299 cells than free EGCG [56], and, in H460 cells, upregulates JWA while downregulating topoisomerase IIα, resulting in diminished invasive capacity [57]. Additionally, EGCG inhibits EMT induced by TGF-β1 through multiple signalling pathways. In A549 cells, EGCG treatment restored glycogen synthase kinase-3β (GSK-3β) activity, increased E-cadherin levels, and reduced *N*-cadherin, vimentin, and Snail expression [58]. EGCG also decreased β-catenin levels and its nuclear localization, while suppressing TGF-β-induced migration, invasion, matrix metalloproteinase 2 (MMP-2) secretion, cell adhesion, and wound healing [58].

EGCG also suppresses migration in A549 cells in a dose- and time-dependent manner, with an IC50 60.55 ± 1.0 μM [59]. Wound healing assays demonstrated that EGCG reduced migration at concentrations around or above 50 μM, further supporting its anti-metastatic potential [59]. Lipid nanoparticles loaded with gemcitabine and EGCG (GEM-EGCG SLNs) inhibited viability of A549 cells with IC50 of 12.5 μg/mL [60]. These nanoparticles were internalized into the nucleus and induced apoptosis through ROS generation, highlighting the potential of nanoformulated EGCG to enhance chemosensitivity and overcome drug resistance [60].

Across lung cancer models, metastatic traits are consistently suppressed by EGCG through inhibition of MMP-mediated invasion and EMT-associated signalling, with downregulation of MMP-2/MMP-9 and restoration of epithelial markers emerging as the most reproducible effects in highly invasive cell lines such as 95-D, CL1-5, A549, and H1299 [50,51,52]. EMT driven by diverse stimuli, including TGF-β-1 and nicotine, is counteracted through interference with Smad-dependent transcription, hypoxia-associated mediators, and EMT-driving transcription factors [53,54,58]. However, the magnitude of anti-migratory effects remains model- and dose-dependent, with stronger responses observed in highly metastatic cells and enhanced efficacy achieved through nanoformulations and combination strategies [56,57,60].

### 2.4. Angiogenesis

Dysregulated angiogenesis is essential for tumour growth and survival. Across lung cancer models, EGCG consistently disrupts angiogenic capacity by suppressing hypoxia-responsive factors, reducing pro-angiogenic cytokine production, and limiting growth-factor-driven endothelial support. A central component of EGCG’s anti-angiogenic activity is its ability to attenuate hypoxia-associated mediators. In A549 and H460 cells, EGCG decreases HIF-1α protein expression, and significantly decreases secretion of VEGF and interleukin-8 (IL-8) at concentrations of 50 µM and 100 µM (Table 2) [65]. Similar reductions in HIF-1α-linked angiogenic output have been observed in HPV-16 E6/E7 models, where EGCG also diminishes Akt activity associated with HIF-1α [65]. Additional evidence shows that EGCG pre-treatment blocks insulin-like growth factor-1 (IGF-1)-stimulated tube-like capillary formation in vitro by suppressing IGF-1-induced HIF-1α and VEGF expression in A549 cells, indicating that EGCG interferes with growth factor-driven angiogenesis [64]. In A549 cells, EGCG simultaneously inhibits cell growth and increases levels of endostatin, while reducing VEGF expression [63]. Pro-angiogenic stimuli associated with tumour-promoting exposures are similarly counteracted by EGCG. In nicotine-treated A549 cells, EGCG dose-dependently suppresses nicotine-induced HIF-1α, VEGF, and COX-2, expression, while restoring β-catenin, and *p53* levels, thereby disrupting multiple pro-angiogenic pathways activated by nicotine exposure [54].

Collectively, reproducible anti-angiogenic effects are observed following EGCG treatment across lung cancer models primarily through suppression of hypoxia-responsive signalling, with downregulation of HIF-1α and VEGF emerging as the most consistent mechanisms in A549, H460, and HPV-16 E6/E7 models [63,64,65]. Angiogenesis driven by diverse stimuli, including IGF-1 and nicotine, is also counteracted, suggesting broad interference with growth factor- and exposure-induced pro-angiogenic pathways [54,64]. However, the magnitude of angiogenic inhibition varies with cell type, EGCG concentration, and experimental context, and most evidence is derived from indirect or in vitro endothelial endpoints. The absence of standardized angiogenesis assays and limited cross-model comparisons constrain direct evaluation of translational relevance, highlighting the need for harmonized angiogenic endpoints in future investigations.

### 2.5. Cancer Stem Cell

Cancer stem cells (CSCs) contribute to tumour initiation, therapeutic resistance, and recurrence. Several studies have shown that EGCG suppresses CSC properties in lung cancer models. Across NSCLC models, EGCG reduces the formation of tumourspheres, indicating impaired self-renewal. In A549 and H1299 cells, EGCG downregulates major CSC markers, including cluster of differentiation 133 (CD133), CD44, aldehyde dehydrogenase 1 family member A1 (ALDH1A1), Nanog, and octamer-binding transcription factor 4 (Oct4), while concurrently suppressing Wnt/β-catenin activity, identifying this pathway as essential for CSC inhibition (Table 2) [33]. Additional work demonstrates that EGCG regulates CSC-associated microRNAs. In A549, H460, and H1299 cells, EGCG increases *miR-485-5p* in a dose-dependent fashion, reversing CSC-like phenotypes induced by suppression of this miRNA, while retinoid X receptor alpha (RXRα) was validated as a direct downstream target [66]. A separate study confirms that EGCG was also able to reverse the CSC-like properties induced by *miR-485* inhibitors in these cells, reinforcing that EGCG suppresses stemness at least in part through miRNA-mediated pathways [36]. Their findings suggested that *miR-485* directly targets CD44, linking EGCG’s molecular effects to disruption of a core CSC regulatory axis [36]. In H1299-sdCSCs EGCG was the most effective catechin at inhibiting sphere formation and reducing *ALDH1A1* and *SNAI2* expression, producing effects comparable to AXL knockdown and implicating AXL receptor tyrosine kinase (AXL)-dependent signalling in EGCG-mediated CSC suppression [67]. EGCG further reduces expression of *CLOCK* mRNA, which is highly expressed in lung CSCs and promotes Wnt/β-catenin signalling and stemness factor expression [68]. EGCG treatment reduces CLOCK expression and decreases the number of CD133^+^ cells, indicating that EGCG disrupts CLOCK-dependent CSC maintenance [68].

Collectively, CSC-associated traits are consistently suppressed by EGCG across multiple NSCLC models, with reduced tumoursphere formation and downregulation of CSC markers representing the most reproducible outcomes [33,36,66,67,68]. Inhibition of Wnt/β-catenin signalling and restoration of tumour-suppressive miRNAs, particularly *miR-485-5p* and *miR-485* targeting RXRα and CD44, respectively, emerge as central mechanisms underlying these effects [33,36,66]. However, CSC inhibition remains model-dependent, with differential reliance on AXL- or CLOCK-associated signalling pathways contributing to variable sensitivity among CSC subpopulations [67,68].

### 2.6. Oncogenic Signalling

Aberrant activation of oncogenic signalling pathways is a major driver of lung cancer progression, and EGCG has been shown to inhibit several key cascades, including EGFR, mesenchymal-epithelial transition factor (c-Met), phosphoinositide 3-kinase/protein kinase B (PI3K/Akt), MAPK, and nuclear factor kappa B (NF-κB). A central target of EGCG is the EGFR axis. EGCG suppresses EGFR phosphorylation in multiple NSCLC cell lines, including A549, H1650, H460, H1975, and HCC927, and inhibits downstream Akt, ERK1/2, and mechanistic target of rapamycin (mTOR) activation (Table 2) [16,37,38]. EGCG blocks EGF-induced activation of EGFR, Akt, ERK1/2, while prolonged exposure reduces EGFR expression and decreases Cyclin D1 levels [16]. Reduced EGFR expression increases resistance to EGCG, indicating that EGCG’s anticancer effects depend in part on EGFR pathway suppression [16]. EGCG also targets c-Met, which is frequently overexpressed in drug-resistant NSCLC. EGCG reduces ligand-induced c-Met phosphorylation and partially inhibits EGFR activation [15]. When combined with EGFR-tyrosine kinase inhibitors (EGFR-TKIs), EGCG suppresses glycolysis, activates AMP-activated protein kinase (AMPK), and inhibits ERK/MAPK, and Akt/mTOR pathways, ultimately inducing cell-cycle arrest and apoptosis in drug-resistant cells [69]. In A549 cells, EGCG reduces phosphorylation of EGFR, Akt, ERK1/2, and mTOR, accompanied by increased apoptosis [37]. In H1299 cells, ECGC induces concentration-dependent reductions in *p*-PI3K and *p*-Akt, while total protein levels remain unchanged [24]. EGCG also inhibits oncogenic signalling through direct interaction with Ras-GTPase-activating protein (GAP)-binding protein 1 (G3BP1). EGCG interact with the Ras-GAP-binding region and glycine-rich domain of G3BP1, leading to reduced Ras-GAP and G3BP1 binding and inhibition of Ras activation [70]. EGCG suppresses anchorage-independent growth in lung cancer cells with high G3BP1 expression, while exhibiting limited effects in cells with low G3BP1 levels [70]. Knockdown of G3BP1 reduced proliferation and anchorage-independent growth and confers resistance to EGCG treatment, indicating a G3BP1-dependent mechanism [70]. EGCG further inhibits downstream G3BP1-mediated mitogen-activated protein kinase kinase (MEK), ERK signalling in G3BP1-expressing cells, with minimal effects observed following G3BP1 depletion [70]. Derivatives of EGCG further strengthen pathway inhibition. An EGCG analog (“compound 3”) combined with cisplatin reduces phosphorylation of EGFR, Akt, and ERK [39], while compounds 11 and 12 (EGCG derivatives containing perbutyrylated glucose residues) similarly decrease phosphorylation of EGFR and downstream signalling proteins [71]. Conjugates of EGCG and EGFR inhibitors enhance these effects, an EGCG-erlotinib conjugate (“compound 10”) binds the extracellular EGFR domain and reduces *p*-EGFR and associated downstream proteins [40]. Dimeric EGCG derivative PBOG (prodelphinidin B-4-3,3’’’-di-O-gallate) similarly inhibited proliferation, migration, and cell-cycle progression in H1975 cells while enhancing apoptosis [41]. Mechanistically, PBOG directly interacted with the EGFR ectodomain, altering its secondary structure, reducing EGFR expression, and suppressing phosphorylation of downstream signalling proteins [41]. EGCG also targets inflammatory and immune-related pathways. EGCG downregulates NF-κB-inducing kinase (NIK), resulting in inhibition of NF-κB [72]. Consistently, EGCG reduces NF-κB nuclear translocation and decreases MMP-9 expression in 95-D cells [50], and lowers MMP-2 levels in CL1-5 cells through JNK inhibition and suppression of NF-κB and specificity protein 1 (Sp1) nuclear translocation [51]. Co-treatment with entacapone or tolcapone reduces NF-κB nuclear translocation [62]. EGCG additionally modulates immune checkpoint signalling by suppressing programmed death-ligand 1 (PD-L1) expression in A549 cells via Janus kinase 2/signal transducer and activator of transcription 1 (JAK2/STAT1) pathway [73], and reduces EGF-induced expression of PD-L1 in Lu99 cells via inhibition of EGFR/Akt signalling [73].

EGCG consistently targets oncogenic signalling in lung cancer, with suppression of EGFR phosphorylation and downstream Akt/ERK/mTOR signalling emerging as the most reproducible and well-validated mechanism across NSCLC models [16,24,37,38,69,70,71]. Inhibition of related receptor tyrosine kinases, including c-Met, and downstream metabolic and survival pathways is most pronounced in EGFR-dependent or drug-resistant contexts, particularly when EGCG is combined with EGFR-TKIs [15,69]. However, EGCG responsiveness remains highly context-dependent, as reduced EGFR expression, low G3BP1 levels, or pathway depletion attenuate its effects, indicating a reliance on intact oncogenic signalling for maximal activity [16,70]. Structural modification and conjugation strategies consistently enhance pathway inhibition and overcome some of these limitations [39,40,41]. Together, these findings indicate that EGCG exerts anti-oncogenic effects primarily through modulation of EGFR-centred signalling networks, but efficacy is strongly influenced by tumour molecular profile and pathway dependency.

### 2.7. Cellular Metabolism and Oxidative Stress

Reprogramming cellular metabolism and resisting oxidative stress are important hallmarks that support lung cancer progression. EGCG interferes with multiple metabolic pathways that sustain tumour growth. EGCG reduces fatty acid synthase activity and expression in A549 cells, while having no effect on carnitine palmitoyltransferase activity (Table 3) [37]. In addition to lipid metabolism, EGCG disrupts glycolytic reprogramming characteristic of the Warburg effect. When combined with *EGFR* tyrosine kinase inhibitors, EGCG suppresses glycolysis, reverses Warburg effect, increases mitochondrial respiration, ROS generation, and reduces the production of lactate in drug-resistant NSCLC cells [69].

EGCG also modulates oxidative stress responses in lung cancer cells. Co-treatment of EGCG with entacapone or tolcapone increases ROS formation [62], consistent with enhanced metabolic stress. The pro-apoptotic effects of EGCG are further enhanced by metabolic-targeting agents. Metformin enhances EGCG-induced ROS levels and suppresses HO-1 expression in A549 and H460 cells, and the combination activates the Nrf2/HO-1 pathway via sirtuin1 (SIRT1)-dependent deacetylation of Nrf2 [18]. Enhanced delivery strategies amplify these effects. EGCG-loaded nanoparticles more effectively inhibit NF-κB activity and its downstream gene expression than free EGCG [42]. EGCG also altered intracellular oxidant levels in invasive lung cancer models. Reduced H_2_O_2_ levels are observed in 95-D cells following EGCG exposure [50].

EGCG increases ROS through CTR1 and nuclear paraspeckle assembly transcript 1 (NEAT) modulation. In A549 and H460 cells, EGCG treatment elevated intracellular ROS by upregulating CTR1 expression [74]. This effect was dose-dependent and involved decreased phosphorylation of ERK1/2 and increased NEAT1 expression, suggesting that NEAT1 acts as an intermediate in EGCG-induced CTR1 upregulation [74].

Collectively, lung cancer metabolism is disrupted by EGCG through inhibition of lipid biosynthesis and glycolytic reprogramming, particularly in metabolically active and drug-resistant NSCLC models [37,69]. These effects are often accompanied by altered ROS levels, with increased oxidative stress reported in combination treatments and CTR1/NEAT1-dependent contexts, while reduced oxidant levels occur in select invasive models [50,62,74]. Nanoformulations further enhance metabolic and redox pathway inhibition [42]. Together, these findings indicate that EGCG exerts context-dependent metabolic and redox effects in lung cancer cells, influenced by baseline metabolic state and treatment combinations.

### 2.8. Epigenetic and Non-Coding RNA

Epigenetic dysregulation and altered non-coding RNA expression contribute to lung cancer development and therapy resistance. Multiple studies demonstrate that EGCG modulates miRNAs, long non-coding RNAs (lncRNAs), and histone-associated processes. EGCG reshapes miRNA expression in a cell line dependent manner (Table 3) [21]. In A549 and LTEP-α-2 cells, EGCG pre-treatment enhances cisplatin-mediated growth inhibition, whereas in H460 cells it increases proliferation and diminishes cisplatin efficacy [21]. These functional differences correspond to differential regulation of *hsa-miR-98-5p*, which is upregulated in H460 cells and downregulated in A549, and inhibition of this miRNA increases cisplatin sensitivity in both cell lines [21]. EGCG also reduces *hsa-miR-125a-3p* expression, and decreased levels of this miRNA promote proliferation in both A549 and H460 cells [21]. Consistent patterns of restored tumour-suppressive miRNA activity have been reported. *hsa-485-5p* was markedly reduced in serum of NSCLC patients and in A549, H460, and H1299 cells and EGCG induces its concentration-dependent upregulation in these cell lines [66]. Inhibition of *hsa-485-5p* increases stemness, but EGCG counteracts these effects, and RXRα is identified as a direct target of the miRNA [66]. Cisplatin-resistant A549 cells similarly display reduced *miR-485* expression and EGCG increases *miR-485* levels while diminishing CD44 in a concentration-dependent manner, with CD44 confirmed as a direct target [36]. EGCG also modulates miRNA expression through HIF-1-dependent mechanisms. In mouse CL13 and human lung cancer H1299, H460, and A549 cells, EGCG upregulates *miR-210* by stabilizing HIF-1 protein and preventing its degradation, leading to increased transcription of the miRNA [75]. Functional studies indicate that these HIF-1/*miR-210*-dependent changes contribute to EGCG-mediated regulation of lung cancer cell behaviour [75]. Beyond miRNAs, EGCG alters 960 lncRNAs and 1434 mRNAs, many of which regulate cell cycle and mitotic cell cycle, and 20 mRNAs are identified as key regulators of EGCG-mediated processes [76]. EGCG also induces broad shifts in miRNA profiles, with distinct set of known and putative miRNAs affected at 40 µM and 100 µM, including those linked to MAPK signalling regulation [14]. EGCG also influences epigenetic regulation at the level of histone modification. Combined treatment with EGCG and Am80 reduces the number of acetylated proteins from 553 to 331, and promotes acetylation of p53 and α-tubulin through suppression of histone deacetylase (HDAC) activity [20]. This combination decreases HDAC4, HDAC5, and HDAC6 protein levels by 20–80% and inhibition of HDAC4 and HDAC5 increases the expression of p21^waf1^, while inhibition of HDAC6 induces the expression of GADD153 and p21^waf1^, contributing to apoptotic signalling [20]. EGCG combined with cisplatin also inhibits the activity of HDAC and DNA methyltransferase (DNMT), and reverses hypermethylation and downregulation of growth arrest-specific 1 (GAS1), tissue inhibitor of metalloproteinase 4 (TIMP4), intercellular adhesion molecule 1 (ICAM1), and Wnt1-inducible signalling pathway protein 2 (WISP2) [22]. EGCG also induces DNA methylation-dependent regulation of Wnt signalling. In A549 and H460 cells, EGCG treatment induces dose-dependent demethylation of Wnt inhibitory factor 1 (*WIF-1*) promoter region, resulting in reactivation of WIF-1 expression [77]. This epigenetic reprogramming is accompanied by reduced cytosolic β-catenin protein levels and suppression of T-cell factor/lymphoid enhancer factor (Tcf/Lef) reporter activity, indicating functional inhibition of Wnt/β-catenin signalling [77].

Collectively, lung cancer epigenetics is modulated by EGCG through regulation of miRNAs, lncRNAs, histone acetylation, and DNA methylation, thereby impacting apoptosis, stemness, and therapeutic response. miRNA expression is altered in a cell line-dependent manner, with enhanced cisplatin sensitivity observed in A549 cells and increased proliferation reported in H460 cells through *hsa-miR-98-5p* regulation [21], while tumour-suppressive miRNAs such as *miR-210* are consistently upregulated through HIF-1-dependent mechanisms [75]. Inhibition of HDAC and DNMT activity, increased p53 acetylation, and demethylation of *WIF-1* further contribute suppression of oncogenic signalling pathways, including Wnt/β-catenin [20,22,77]. The majority of studies support EGCG’s anticancer activity via these epigenetic and non-coding RNA mechanisms, with only a single study (by Zhou et al., 2014) reporting a context-specific proliferative effect [21]. Although these epigenetic effects are generally robust, they remain context-dependent and are influenced by cell variability and treatment combinations, highlighting challenges for clinical translation.

### 2.9. Immune Modulation

Immune evasion and dysregulated inflammatory signalling are central features of lung cancer progression. EGCG has been shown to modulate several immune regulatory pathways, particularly those involving immune checkpoint control, cytokine-associated signalling, and NF-κB-mediated transcription. Multiple studies highlight a consistent inhibitory effect of EGCG on PD-L1 expression. EGCG inhibits interferon-γ (IFN-γ) and EGF-induced PD-L1 protein expression in A549 and H1299 cells (Table 3) [73]. Pre-treatment with EGCG and green tea extract reduces IFN-γ-induced *PD-L1* mRNA and protein expression by 40–80% in A549 cells through inhibition of the JAK2/STAT1 pathway [73]. EGCG also blocks EGF-induced expression of PD-L1 in Lu99 cells by 37–50% via inhibition of EGFR/Akt pathway [73]. EGCG further disrupts inflammatory signalling networks associated with angiogenic and cytokine regulatory pathways. EGCG inhibits HIF-1α protein expression induced by HPV-16 E6 and E7 in A549 and H460 cells, resulting in reduced secretion of IL-8 and VEGF [65]. EGCG also blocks HIF-1α-driven Akt [65]. Additional immune-related regulation is observed in cisplatin resistant and parental NSCLC cells, where EGCG increases IL-6 production without altering STAT3 phosphorylation, suggesting that IL-6/STAT3 signalling does not mediate the effects of EGCG [61]. EGCG treatment also downregulates Axl and tyrosine-protein kinase receptor TYRO3 (Tyro3) protein and mRNA levels in these cells [61]. EGCG exerts broader immunomodulatory effects through inhibition of NF-κB and related transcriptional regulators. EGCG combined with okadaic acid downregulates NIK in NSCLC cells, a key upstream activator of the NF-κB activation [72]. Co-treatment of EGCG with entacapone or tolcapone reduces NF-κB translocation to the nucleus in H1299 and CL-13 cells [62]. EGCG combined with metformin suppresses HO-1 expression in A549 and H460 cells [18]. This combination activates the Nrf2/HO-1 pathway via SIRT1-dependent deacetylation of Nrf2, and SIRT1 expression was partly mediated by NF-κB pathway [18]. Enhanced inhibition of NF-κB activity is also achieved through nanoparticle delivery of EGCG, which more effectively suppresses NF-κB-regulated gene expression than free EGCG [42]. Similarly, EGCG acts synergistically with the NF-κB inhibitor BAY11-7082 to suppress proliferation in A549 and H1299 cells [43].

Collectively, lung cancer immunity is modulated by EGCG through downregulation of PD-L1 and suppression of inflammatory signalling pathways, including JAK/STAT, EGFR/Akt, and NF-κB. Inhibition of IFN-γ- and EGF-induced PD-L1 expression via JAK2/STAT1 and EGFR/Akt suppression is consistently observed across A549, H1299, and Lu99 cells [73], while reduction in HIF-1α-driven IL-8 and VEGF secretion links immune modulation to anti-angiogenic effects [65]. EGCG also downregulated TAM receptors (Axl, Tyro3), enhanced IL-6 without activating STAT3, and suppressed NF-κB via multiple upstream regulators, with effects potentiated by combination treatments, metformin, or nanoparticle delivery [18,42,43,61,62,72]. However, variability in immune effects is influenced by cell type, cytokine milieu, and tumour context, and clinical translation remains constrained by pharmacokinetic limitations and limited systemic evaluation.

### 2.10. Drug Resistance

Drug resistance is a major hurdle in lung cancer therapy. EGCG combined with cisplatin suppressed the proliferation of A549 cells in a concentration-dependent manner, and pre-treatment with EGCG for four hours produced the greatest inhibitory effect in A549 and LTEP-α-2 cells (Table 3) [21]. In contrast, EGCG enhanced proliferation in H460 cells and reduced the efficacy of cisplatin [21]. They discovered that *hsa-miR-98-5p* was upregulated in H460 cells and downregulated in A549 cells, and inhibition of this miRNA increased cisplatin sensitivity in both cell lines [21]. *hsa-miR-125a-3p* was reduced in both cell lines after EGCG exposure, with a more pronounced decrease in H460 cells, and suppression of this miRNA promoted proliferation of A549 and H460 cells [21]. Combination of EGCG and cisplatin reduced proliferation, increased G1 arrest, and enhanced apoptosis in cisplatin-resistant A549 cells [22]. Treatment inhibited HDAC and DNMT activity and reversed hypermethylation and downregulation of GAS1, TIMP4, ICAM1, and WISP2 [22]. EGCG reduced proliferation and colony formation of the cisplatin-resistant variants A549 and H460 variants and their parental lines in a concentration-dependent manner [61]. Treatment increased IL-6 production, but did not alter STAT3 phosphorylation, indicating that IL-6/STAT3 signalling were not involved in mediating the response [61]. EGCG decreased Axl and Tyro3 protein, and mRNA levels in both cell lines [61]. EGCG also improves the efficacy of targeted therapies in drug-resistant models. Combination of EGCG with EGFR-TKIs suppressed glycolysis, reversed Warburg effect, increased mitochondrial respiration, ROS generation, and decreased the production of lactate in drug-resistant NSCLC cells [69]. This combination activated AMPK and inhibited ERK/MAPK and Akt/mTOR signalling, leading to cell-cycle arrest and apoptosis specifically in drug-resistant cells [69]. Structural analogues further highlight the role of EGCG in modulating resistance. EGCG derivative “compound 3” inhibited the proliferation and colony formation, and induced cell-cycle phase redistribution and apoptosis in H441 cells [39]. Combined treatment with cisplatin produced stronger inhibitory effects including modulation of PCNA, *p*-p53, Bax, and Bcl-2 [39]. The combination also reduced phosphorylation of EGFR, Akt, and ERK [39]. Another analog, “compound 10”, inhibited proliferation, migration, and colony formation, while increasing apoptosis in H1975 cells and induced cell-cycle arrest [40]. This compound bound to the extracellular domain of EGFR, and reduced *p*-EGFR and downstream signalling [40]. Sequential chemotherapy combinations further potentiate EGCG’s anticancer effect. Sequential treatment of H460 cells with paclitaxel followed by EGCG resulted in synergistic suppression of cell growth [44]. Paclitaxel first, then EGCG, significantly inhibited Bcl-2 and procaspase-3 while increasing cleaved PARP levels, indicating enhanced apoptosis [44]. EGCG enhances the efficacy of additional chemotherapeutic agents. In A549, H358, and H1975 cells, EGCG inhibited cell growth in a time- and dose-dependent manner [45]. Co-treatment with EGCG increased the effectiveness of 5-fluorouracil (5-FU) and doxorubicin in suppressing cell growth and inducing apoptosis [45]. Furthermore, EGCG reduced ERK phosphorylation in a dose-dependent manner and further suppressed ERK activation induced by gemcitabine, 5-FU, and doxorubicin, demonstrating its potential to improve chemosensitivity [45]. Low-dose EGCG further enhanced doxorubicin sensitivity in A549 cells by reducing drug efflux, inhibiting multidrug resistance signalling, and decreasing invasiveness, while increasing drug uptake, promoting cell-cycle arrest, including inducing stress-related damage, and ultimately triggering cells’ death [78]. Resistance in A549 cells was attributed to persistent Nrf2 activation, which EGCG modulated in a pro-oxidative manner [78]. EGCG combined with gefitinib similarly overcomes drug resistance. In gefitinib-resistant A549 cells, co-treatment increased apoptosis, as shown by TUNEL-positive cells, and reduced proliferation, indicated by decreased Ki-67 staining [46]. The combination inhibited autophagic flux by decreasing ATG5 and LC3 II/I levels and increasing p62 expression [46]. Additionally, EGCG suppressed gefitinib-induced activation of the rapidly accelerated fibrosarcoma (Raf)/MEK/ERK signalling pathway, demonstrating that it can overcome gefitinib resistance by simultaneously promoting apoptosis, inhibiting autophagy, and targeting key cell-survival signalling [46]. EGCG further improves the antitumour efficacy of combination therapies. In H1299 cells, co-treatment with EGCG and apatinib decreased viability, proliferation, migration, invasion, and glycolysis, while increasing apoptosis [47]. Mechanistically, EGCG regulated glycolysis through VEGF overexpression, enhancing the antitumour effects of apatinib [47].

Collectively, EGCG modulates drug resistance in lung cancer by sensitizing NSCLC cells to chemotherapeutics and targeted agents, although effects are highly cell line- and context-dependent. Cisplatin efficacy is enhanced in A549 and LTEP-α-2 cells through G1 arrest, apoptosis, and epigenetic reprogramming, whereas only one study reported paradoxical proliferative effects in H460 cells (in a study by Zhou et al., 2014) [21]. EGCG additionally inhibits survival signalling, reverses the Warburg effect, downregulates Axl/Tyro3, and modulates autophagy and ROS, thereby improving response to EGFR-TKIs and chemotherapeutics such as 5-FU, doxorubicin, and gemcitabine [44,45,46,47,61,69,78]. Derivatives and combination strategies further enhance antiproliferative and pro-apoptotic effects [39,40]. Although these resistance-modifying effects are reproducible in preclinical models, they are highly context-specific, and clinical translation remains limited by pharmacokinetics and the absence of trials targeting resistance.

## 3. In Vivo Anticancer Effects of EGCG

Multiple in vivo studies demonstrate that EGCG suppresses lung cancer development, progression, and tumour growth through diverse mechanisms. In a tobacco-specific nitrosamine (NNK)-induced lung cancer (11.65 mg/kg body weight, three times per week for 10 weeks) mouse model, EGCG (560 ppm in drinking water for 13 weeks) reduced tumour formation and inhibited 8-hydroxydeoxyguanosine (8-OH-dGuo) accumulation in lung DNA (Table 4) [79]. In the same carcinogen-driven model, dietary EGCG at 0.4% for one week altered the expression of twenty-one microRNAs in lung tumours, with the most affected pathways involving Akt, NF-κB, MAP kinases, and cell-cycle regulation [80]. Similarly, A/J mice receiving EGCG (1 mg/mL in drinking water) in combination with cisplatin (1.62 mg/kg/week) exhibited fewer tumours and reduced cisplatin-induced body weight loss compared with cisplatin alone [12]. EGCG also enhanced the efficacy of targeted therapies. In SCID mice bearing H460 xenografts, EGCG (15 mg/kg) combined with erlotinib (10 mg/kg) significantly decreased tumour weight and volume 22 days post-implantation [15]. Polyphenon E (Poly E, 1.5% wt/wt in diet) and EGCG (0.525% wt/wt) enhanced the stability of EGCG in B(*a*)P-induced tumour model [81]. EGCG in combination with luteolin (125 mg/kg and 20 mg/kg, respectively) markedly suppressed tumour growth over 40 days [27]. Dose-dependent inhibition of tumour growth and increased markers of apoptosis (cleaved caspase-3), oxidative stress (8-OHdG), DNA damage-induced repair (γ-H2AX) were observed in mice treated at 0.1–0.5% in diet or 30 mg/kg intraperitoneally [17]. Daily intraperitoneal administration of EGCG (50 mg/kg) for two weeks induced apoptosis in vivo, accompanied by increased cleaved caspase-3 and upregulation of *Bax* at both protein and mRNA levels, while Bcl-xL and Ku70 expression were reduced, and the Ku70-Bax interaction was disrupted that EGCG could interrupt the interaction between the two [82]. Intraperitoneal EGCG (50 mg/kg, twice weekly) reduced the number of lung tumour nodules [51], and at 40 mg/kg weekly induced PARP cleavage and apoptosis without affecting fatty acid synthase expression [37]. In additional xenograft studies, EGCG administered intraperitoneally at 20 mg/kg twice weekly for four weeks significantly inhibited tumour growth while reducing topoisomerase IIα protein levels and increasing JWA expression [57]. In nicotine-exposed models, EGCG inhibited HIF-1α-dependent angiogenesis in vivo, and downregulated HIF-1α and VEGF expression in A549 xenografted nude mice after eleven days [54]. EGCG also demonstrated anti-angiogenic effect in vivo, reducing HIF-1α, VEGF, CD31, and IGF-induced hemoglobin levels, and CD34-positive vessels in multiple models [63,64,65]. Combination therapies enhanced these effects, as seen with metformin, BAY11-7082, or EGCG nanoparticles [18,42,43]. In carcinogen-induced lung cancer models, oral administration of green tea extract 0.3% in drinking water for sixteen weeks reduced tumour multiplicity and significantly decreased the proportion of PD-L1 positive cells, supporting an immunomodulatory role for EGCG-containing formulation in vivo [73].

EGCG further modulated tumour proliferation, apoptosis, and chemoresistance. In A549 xenografts, EGCG enhanced cisplatin efficacy via regulation of *hsa-miR-98-5p* and CTR1 [32]. Consistent with this mechanism, intraperitoneal EGCG at 20 mg/kg administered every three days for two weeks increased ROS generation and upregulated NEAT1 and CTR1 expression while reducing ERK1/2 phosphorylation in vivo [74]. Nanoformulations and derivatives (e.g., compound 3, EGCG-NPs, PBOG) improved tumour suppression, reduced Ki67, *p*-EGFR, *p*-Akt, *p*-ERK, and increased apoptosis [39,41,42,60]. EGCG also targeted cancer stem-like cells in vivo, reducing CD133, CD44, Sox2, Nanog, Oct4, and CLOCK expression, while enhancing *hsa-miR-485-5p* levels, suppressing CSC properties [36,66,68]. Consistent with these findings, oral administration of EGCG at 100 mg/kg or green tea extract at 0.2% in drinking water suppressed tumour growth derived from AXL-high H1299 clones and reduced *p*-AXL, ALDH1A1, and SLUG expression in vivo [67].

Additional studies demonstrated EGCG’s efficacy in diet- and carcinogen-induced models, high-fat diet combinations, and its capacity to improve survival and reduce tumour progression in combination with cisplatin, metformin, gefitinib, apatinib, tolcapone, osimertinib, and curcumin [12,19,21,22,46,47,49,69].

EGCG suppresses lung cancer development and tumour growth in vivo through induction of apoptosis, reduction in proliferation, inhibition of angiogenesis, and targeting of cancer stem-like properties. These effects are enhanced by combination strategies with cisplatin, erlotinib, luteolin, metformin, or nanoparticle formulation, resulting in increased cleaved caspase-3, Bax, and ROS, and reduced Bcl-xL, Ki-67, oncogenic signalling (*p*-EGFR, *p*-Akt, *p*-ERK), and PD-L1 expression [12,15,17,18,19,21,22,27,32,39,41,42,43,46,47,49,57,60,63,64,65,69,73,74]. The magnitude of tumour inhibition varies across models, with relatively stronger suppression observed in xenografts and combination regimens and more modest effects in carcinogen-induced models, reflecting context-dependent outcomes influenced by miRNA regulation (e.g., *hsa-miR-98-5p*) [32,80]. These findings indicate reproducible anti-tumour activity of EGCG in preclinical lung cancer models, while highlighting model dependence and pharmacokinetic limitations that constrain clinical translation.

## 4. Pharmacokinetics and Bioavailability

Although many studies have reported the anticancer effects of EGCG and green tea polyphenols, only a small number of studies have explicitly examined their bioavailability in humans or preclinical models, and the majority of human studies were not conducted in lung cancer patients.

In mice, prostate EGCG concentrations 0.24 nmol/g were significantly reduced within 24 h of tea withdrawal, indicating limited tissue retention, whereas in men consuming green or black tea for five days prior to prostatectomy, prostate tissue EGCG concentrations of 40 ± 11 and 66 ± 10 pmol/g for green tea and black tea, respectively, were found [83]. Following single-dose intravenous administration of EGCG at 21.8 µmol/kg in mice (30–35 g), peak plasma concentrations of 2.7 ± 0.7 µM were observed, whereas intragastric administration at a higher dose 163.8 µmol/kg yielded lower plasma concentrations of 0.28 ± 0.08 µM, with 50–90% of EGCG present as glucuronide conjugates [84]. Unconjugated EGCG was detected in lung and prostate tissue at 0.31–3.56 nmol/g after intravenous dosing, while intragastric administration resulted in substantially lower tissue levels [84]. In the small intestine, EGCG levels were 45.3 ± 13.5 nmol/g, whereas colonic levels were 7.86 ± 2.4 nmol/g [84]. Single-dose intragastric EGCG 50–2000 mg/kg in mice produced dose-dependent plasma and tissue levels, with linear increases observed in plasma 0.03–4.17 µg/mL (0.065–9.1 µM), prostate 0.01–0.91 µg/g (approximately 0.022–1.98 µM), and liver 0.09–18.3 µg/g (approximately 0.20–39.9 µM) [85]. In contrast, small intestinal and colonic tissue levels plateaued at high doses, indicating saturation [85].

In healthy volunteers, in a single ascending dose study of 94% pure EGCG, rapid absorption was observed with peak plasma levels occurring 1.3–2.2 h across 50–1600 mg [86]. Maximum mean total EGCG concentrations in plasma ranged from 130.37 to 3391.60 ng/mL (0.28–7.40 µM), while maximum mean free EGCG concentrations ranged from 119.83 to 2911.35 ng/mL (0.266–6.35 µM), and elimination half-lives ranged from 3.9 to 5.5 h [86]. Repeated dosing over 10 days in healthy volunteers at 200–800 mg/day was well tolerated, with generally dose-linear plasma kinetics at 200–400 mg, and slightly more than dose-proportional exposure at 800 mg [87]. This pattern indicated partial saturation of clearance pathways, with elimination half-lives of 2.7–3.4 h on the first day and 2.3–5.2 h on day ten [87]. In another single-dose study, ingestion of decaffeinated green tea (1.5–4.5 g), resulted in peak plasma EGCG concentrations of up to ~326 ng/mL (approximately 0.71 µM) at 1.4–2.4 h; plasma levels were increased when the dose was raised from 1.5 to 3.0 g but did not increase further at 4.5 g, suggesting a plateau in systemic exposure [88]. A half-life of ~5–5.5 h was observed, and EGCG was not detected in urine [88]. Four weeks of oral administration of 800 mg once daily in humans resulted in plasma levels of 234.9 ± 140.9 ng/mL (approximately 0.51 ± 0.31 µM) on the first day and 390.3 ± 231.4 ng/mL (approximately 0.85 ± 0.51 µM) on the last day [89]. These trends were confirmed in additional studies in healthy volunteers, in which single oral doses produced peak plasma concentrations in the micromolar range [90,91,92,93]. Across studies, EGCG exhibited elimination half-lives of 3.4–3.9 h, negligible urinary excretion, minimal conversion to EGC, and substantial interindividual variability [90,92]. Pharmacokinetic profiles were similar whether EGCG was administered as a pure compound or as part of green tea [93]. In a presurgical study of early breast cancer patients, daily supplementation with a lecithin-formulated green tea extract providing 44.9 mg EGCG for four weeks resulted in EGCG being detected in 100% of tumour tissue samples, with total concentrations reaching up to 8 ng/g (approximately 0.0174 µM) [94]. Median EGCG levels were higher in tumour tissue than in adjacent normal tissue (0 ng/g), and plasma-free EGCG concentrations 8–65.8 ng/mL (0.017–0.144 µM) were correlated with reduced tumour proliferation, demonstrating improved bioavailability and tissue delivery with the formulated preparation [94].

Despite demonstrated antioxidant and anticancer potential, EGCG exhibits limited and inconsistent oral bioavailability, which complicates translation of in vitro efficacy to clinical outcomes [95]. Nevertheless, measurable EGCG concentrations have been observed in plasma, with free plasma levels ranging from 8 to 2911.35 ng/mL (0.017–6.35 µM). Factors contributing to this limitation include EGCG’s physicochemical properties, instability, low absorption, and extensive metabolism [95]. Various strategies have been proposed to enhance systemic exposure, including co-administration with foods, formulation approaches such as emulsions or nanoparticles, and structural modification of the EGCG molecule [95].

## 5. Clinical Evidence of EGCG in Lung Cancer

Clinical evidence supporting the direct anticancer efficacy of EGCG in lung cancer remains limited, with available studies being primarily focused on safety and supportive-care outcomes rather than tumour control. Dosages for each study are summarized in Table 5, and the observed clinical benefits were limited to supportive-care outcomes, such as reducing radiation-induced esophagitis and associated symptoms, rather than direct effects on tumour control or survival. To date, no randomized control trials have demonstrated a direct effect of EGCG on lung tumour regression, progression-free survival, or overall survival. A phase I clinical trial was conducted to evaluate the safety and tolerability of orally administered EGCG in patients undergoing thoracic radiotherapy [96]. Across six escalating dose levels (40, 80, 140, 210, 300, and 440 μmol/L), EGCG was well tolerated, and no dose-limiting toxicities were observed, indicating a favourable safety profile in this clinical context [96]. Importantly, the primary clinical benefit observed was a significant reduction in radiation-induced esophagitis, with 22 to 24 patients experiencing only grade 0–1 esophagitis during treatment [96]. In parallel, reduced pain severity was reported, indicating a potential protective and symptom-relieving effect of EGCG during radiotherapy rather than a direct anticancer effect [96].

These findings were further supported by a subsequent phase II clinical trial, in which a reduction in the incidence and severity of acute radiation-induced esophagitis was demonstrated following oral EGCG (440 μmol/L) administration in patients receiving thoracic radiotherapy [97]. Significantly lower maximum esophagitis grades, along with reduced pain and dysphagia scores, were observed in patients receiving prophylactic or therapeutic oral EGCG (440 μmol/L) during chemoradiation compared with conventional therapy [98]. Slightly greater protection was observed with prophylactic EGCG administration compared with therapeutic use, and no significant side effects were reported, demonstrating the safety and efficacy of EGCG for mitigating acute radiation-induced esophagitis [98]. However, tumour response, disease progression, and long-term oncologic outcomes were not evaluated in the trials, limiting conclusions regarding EGCG’s role as a therapeutic anticancer agent in lung cancer.

Collectively, very limited current clinical data indicate that EGCG may be a safe adjunctive agent with potential benefits in mitigating treatment-related toxicity, particularly radiation-induced esophagitis. While these findings are clinically relevant, they do not provide evidence for direct tumour control or disease-modifying activity in lung cancer patients. Translation of the robust preclinical anticancer effects of EGCG to clinical oncology will require well-designed trials incorporating pharmacokinetic optimization and clinically meaningful oncologic endpoints.

## 6. Conclusions

**Figure 2:**
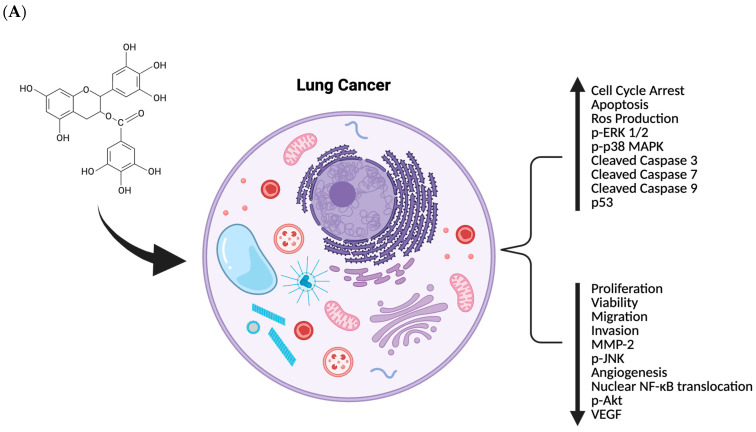

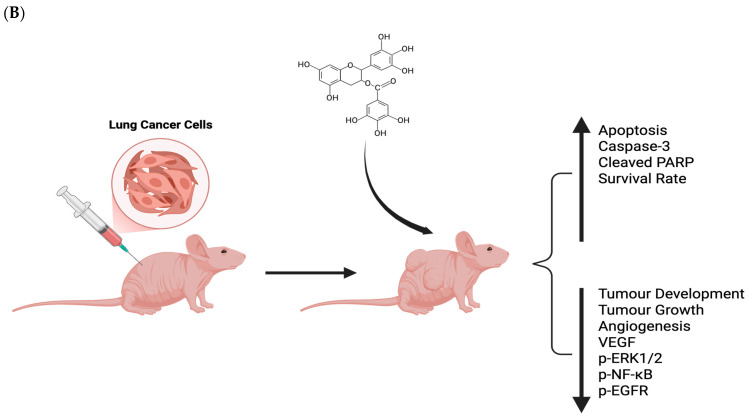
Summary of in vitro (**A**) and in vivo (**B**) effects of epigallocatechin gallate against lung cancer. Created in BioRender. Dordaneh Mirbabaei Ghafghazi. (2026) https://www.biorender.com.

This review provides a comprehensive and critical synthesis of the anticancer effects of epigallocatechin gallate (EGCG) in lung cancer, integrating evidence from in vitro, in vivo, and limited clinical studies encompassing both earlier seminal studies and recent advances reported between 2020 and 2024. Collectively, preclinical data demonstrate that EGCG targets multiple hallmarks of lung cancer, including proliferation, apoptosis and ferroptosis, migration and metastasis, angiogenesis, cancer stemness, oncogenic signalling, metabolic reprogramming, immune evasion, and drug resistance (Figure 2). Among these mechanisms, induction of apoptosis, suppression of EGFR-related signalling, inhibition of epithelial–mesenchymal transition, and modulation of oxidative stress emerge as the most reproducible effects across diverse lung cancer models.

Importantly, this review distinguishes between EGCG responses that are consistently observed across models and those that are highly dependent on cell lines, genetic background, molecular context, and treatment conditions. One study reported proliferative effects, underscoring the need for cautious interpretation and reinforcing the importance of tumour heterogeneity in determining EGCG responsiveness. While combination strategies, nanoformulations, and molecular conjugates generally enhance efficacy and overcome some resistance mechanisms, many reported effects at concentrations that exceed clinically achievable plasma levels.

Clinical evidence to date supports EGCG primarily as a safe adjunctive agent capable of reducing treatment-relate toxicity, particularly radiation-induced esophagitis, rather than as a standalone anticancer therapy. The absence of trials evaluating tumour response or survival endpoints highlights a critical translation gap.

Overall, this review provides a comparative and mechanistically grounded evaluation of EGCG’s anticancer potential in lung cancer. Future progress will depend on pharmacokinetic optimization and rigorously designed clinical studies to determine whether EGCG can be effectively integrated into lung cancer prevention or treatment strategies.

## 7. Limitations and Future Directions

Despite extensive preclinical evidence, several limitations constrain the translational relevance of EGCG in lung cancer. First, many studies rely on in vitro models and employ EGCG concentrations of 20–100 µM that are higher than plasma levels typically achieved following oral administration. In contrast, in vivo studies demonstrate that EGCG plasma concentrations remain much lower, ranging from approximately 0.065–9.1 µM in animal models and 0.017–6.35 µM for free EGCG in humans. While such experimental approaches provide valuable mechanistic insights, they may limit direct translational relevance and highlight the need for careful interpretation in a clinical context. Second, EGCG exhibits poor oral bioavailability and rapid metabolism, resulting in low plasma and tissue concentrations relative to reported in vitro IC50 values. Third, substantial heterogeneity exists across lung cancer models, with cell line-specific genetic and metabolic differences leading to variable responses to EGCG treatment. Although only one study has reported contradictory results, these findings may reflect limitations related to specific experimental models or conditions. Nevertheless, further well-controlled studies are needed to fully clarify the contexts in which EGCG exhibits anticancer activity, as the majority of evidence supports its efficacy. Future studies should prioritize pharmacokinetic and pharmacodynamic optimization, including advanced delivery systems such as nanoformulations and conjugates, to improve bioavailability and tumour targeting. Comparative studies across molecularly defined lung cancer subtypes are needed to identify biomarkers predictive of EGCG sensitivity. In addition, clinical trials should move beyond safety and supportive-care endpoints to evaluate tumour response, disease progression, and survival outcomes, ideally in combination with standard chemotherapeutic, targeted, or immunotherapeutic agents. Addressing these limitations will be essential to determine the true therapeutic potential of EGCG in lung cancer management.

## Figures and Tables

**Table 1:** Mechanistic summary of EGCG effects on lung cancer: proliferation and cell-cycle, apoptosis and ferroptosis, migration, invasion, and EMT.

**Proliferation and Cell-Cycle Control**			
**Cell Lines**	**Treatment**	**Findings**	**References**
PC-9, H1299, H441, A549, H1650, H1975, H460, Hcc827, H358, SBC-3, H69 (drug-sensitive and drug-resistant variant variants), LK-2	EGCG ± chemotherapy or targeted agents, or nanoformulations/derivatives	↓ proliferation, colony formation, anchorage-dependent growth, Ki67, PCNA; **↑** cell-cycle arrest G0/G1, S, and G2/M phases; ↓ Cyclin D1, Cyclin B1; **↑** cell-cycle inhibitors (p21^Cip1/Waf1^); effective in therapy-resistant and parental NSCLC cells; enhanced cell-cycle arrest in combination treatments	[10,11,12,13,14,15,16,17,18,19,20,21,22]
**Apoptosis and Ferroptosis**			
**Cell Lines**	**Treatment**	**Findings**	**References**
H661, HCC827, H358, H1975, H292, Calu-1, PC-9, H441, SPC-A-1, A549, H1299, H460, cisplatin-resistant A549, gefitinib-resistant A549, H69 drug-sensitive and drug-resistant variants	EGCG ± chemotherapy, TKIs, derivatives, or nanoformulations	**↑** apoptosis, **↑** intrinsic and extrinsic apoptotic pathways (**↑** cytochrome c, DR5, **↑** caspase cascade activation (caspase-3, -7, -8, -9, cleaved PARP, DNA fragmentation); p53-dependent apoptosis (**↑** Ser15 phosphorylated p53, mitochondrial translocation); Bcl-2 family modulation (**↑** Bax; ↓ Bcl-2, Bcl-xL; Ku70 acetylation leads to Bax release); **↑** pro-apoptotic stress markers (GADD153); enhanced chemotherapy induced apoptosis	[11,17,18,20,22,23,24,25,26,27,28,29,30,31,32,33,34,35,36,37,38,39,40,41,42,43,44,45,46,47]
A549, H1299	EGCG	↓ *GPX4*, *SLC7A11*, *tsRNA-13502*; leptin-induced cell survival, colony formation, migration, invasion (through *STAT1*/*SLC7A11*) **↑** *ACSL4*, MDA, ROS, ferroptosis leptin-induced cell survival, colony formation, migration, invasion	[48,49]
**Cell Migration, Invasion and EMT**			
**Cell Lines**	**Treatment**	**Findings**	**References**
CL1-5, A549, H1299, H1975, 95-D, Lu99	EGCG alone, nanoformulations/derivatives, or combined with chemotherapeutics, TKIs, or pathway inhibitors	↓ invasion, migration; ↓ MMP-2/MMP-9; ↓ vimentin, *N*-cadherin, Snail, Slug, Zeb1, Twist1; inhibition of TGF-β-induced EMT; ↓ Smad2, ERK, Akt, EGFR, NF-κB signalling; ↓ β-catenin nuclear localization; **↑** E-cadherin expression; reduced wound healing and cell motility	[21,28,35,38,40,41,43,47,49,50,51,52,53,54,55,56,57,58,59,60,61,62,63,64]

**Table legend:** ↑ Increased, ↓ reduced.

**Table 2:** Mechanistic summary of EGCG effects on lung cancer: angiogenesis, cancer stem cells, and oncogenic signalling.

**Angiogenesis**			
**Cell Lines**	**Treatment**	**Findings**	**References**
A549	EGCG ± IGF-1	↓ angiogenesis; ↓ HIF-1α, VEGF, IL-8, COX-2, and Akt/ERK signalling; inhibition of IGF-1- and HPV- induced tube formation; ↑ endostatin expression	[54,63,64,65]
**Cancer Stem Cells**			
**Cell Lines**	**Treatment**	**Findings**	**References**
A549, H1299, cisplatin-resistant A549, H460	EGCG ± *hsa-miR-485-5p*	↓ CSC markers and properties (CD133, CD44, *ALDH1A1*, Nanog, Oct4, CLOCK); ↓ tumoursphere formation and CSC-like properties; ↑ *hsa-miR-485-5p*; suppression of stemness-associated transcriptional programmes	[33,36,66,67,68]
**Oncogenic Signalling**			
**Cell Lines**	**Treatment**	**Findings**	**References**
H2122, H358, A549, H1650, H460, H1299, CL13, 95-D, CL1-5, HCC827, H1975, gefitinib- or osimertinib-resistant H1975, H441, PC-9, Lu99	EGCG ± targeted agents, chemotherapy, or derivatives	↓ EGFR activation and phosphorylation; inhibition of downstream Akt, ERK/MAPK, mTOR, PI3K, and p38 MAPK signalling; suppression of c-Met activation; reduced Ras-MEK-ERK signalling; inhibition of NF-κB nuclear translocation and JNK signalling	[15,16,24,37,38,39,40,41,50,51,62,69,70,71,72,73]

**Table legend:** ↑ Increased, ↓ reduced.

**Table 3:** Mechanistic summary of EGCG effects on lung cancer: cellular metabolism and oxidative stress, epigenetic and non-coding RNA, immune modulation, and drug resistance.

**Cellular Metabolism and Oxidative Stress**			
**Cell Lines**	**Treatment**	**Findings**	**References**
A549, gefitinib- or osimertinib-resistant H1975, H1299, CL-13, H460, 95-D	EGCG ± gefitinib/osimertinib, metabolic modulators, or nanoparticles	↓ glycolysis, Warburg effect, and lactate production; ↓ fatty acid synthase activity; modulation of Akt/mTOR-linked metabolic signalling; ↑ mitochondrial respiration, AMPK activation, and intracellular ROS; regulation of redox pathways via Nrf2/HO-1 suppression; altered antioxidant and stress-response signalling	[18,37,42,50,62,69,74]
**Epigenetic and Non-Coding RNA**			
**Cell Lines**	**Treatment**	**Findings**	**References**
A549, PC-9, LTEP-α-2, H460, H1299, cisplatin-resistant A549, CL13	EGCG ± cisplatin, retinoids (Am80), or miRNA modulation (*miR-210*, *miR485-5p*)	↓ HDAC activity and HDAC 4/5/6; ↓ DNMT activity; ↓ β-catenin expression; demethylation of *WIF-1* promoter; ↑ acetylation of p53, α-tubulin, and multiple proteins; modulation of miRNAs (*hsa-miR-98-5p*, *miR-125a-3p*, *miR-485-5p*, *miR-210*) and long non-coding RNAs; altered expression of mRNAs (*GAS1*, *TIMP4*, *ICAM1*, *WISP2*)	[14,20,21,22,36,66,75,76,77]
**Immune Modulation**			
**Cell Lines**	**Treatment**	**Findings**	**References**
A549, H1299, Lu99, H460, cisplatin-resistant H460, PC-9, CL-13	EGCG ± metabolic or signalling inhibitors; nano-EGCG formulations	↓ PD-L1 expression (IFN-γ/EGF-induced) ↓ Axl and Tyro3 ↓ NF-κB activity and nuclear translocation; ↑ IL-6 production and STAT3; ↓ IL-8 production	[18,42,43,61,62,65,72,73]
**Drug Resistance**			
**Cell Lines**	**Treatment**	**Findings**	**References**
A549, LTEP-α-2, H460, gefitinib- or osimertinib-resistant H1975, H441, H1975, cisplatin resistant A549 and H460, H358, gefitinib-resistant A549, H1299	EGCG ± chemotherapy or targeted agents (cisplatin, doxorubicin, 5-FU, gemcitabine, paclitaxel, gefitinib, osimertinib, erlotinib, apatinib), or EGCG derivative	↓ proliferation, colony formation, migration, invasion, drug efflux, multidrug resistance signalling, glycolysis, ERK/MAPK, Akt/mTOR, EGFR, Axl, Tyro3, Bcl-2, procaspase-3, autophagic flux, ATG5, LC3 II/I; ↑ apoptosis, cell-cycle arrest, cleaved PARP, Bax, p53 activation, ROS production, mitochondrial respiration, AMPK activation, enhanced uptake of chemotherapeutics	[21,22,39,40,44,45,46,47,61,69,78]

**Table legend:** ↑ Increased, ↓ reduced.

**Table 4:** Mechanistic summary of EGCG effects in lung cancer: in vivo findings.

In Vivo Anticancer Effects of EGCG			
Model	Treatment	Findings	References
NNK- and B(*a*)P-induced lung cancer; A/J mice	Dietary EGCG or polyphenon E	↓ tumour formation; ↓ oxidative DNA damage (8-OH-dGuo); miRNA reprogramming affecting Akt, NF-κB, MAPK, and cell-cycle pathways; improved EGCG stability in Poly E formulations	[79,80,81]
A/J mice; H460 and A549 xenografts	EGCG ± cisplatin, erlotinib, luteolin	↓ tumour burden, weight, and volume; enhanced efficacy of chemotherapy and EGFR-targeted therapy; reduced treatment-associated toxicity	[12,15,27]
Xenografts (H1299, CL1-5, A549)	Dietary or intraperitoneal EGCG	Dose-dependent ↓ tumour growth; ↑ apoptosis (cleaved caspase-3, Bax, PARP cleavage); ↑ oxidative stress (8-OHdG); ↑ DNA damage-induced repair (γ-H2AX); ↓ Bcl-xL and; ↓ topoisomerase IIα; ↑ JWA	[17,43,51,57,82]
Nicotine-, IGF-1-, HPV-E6/E7-driven and A549-driven xenografts	EGCG	↓ HIF-1α-dependent angiogenesis; ↓ HPV-16 E6- and E7-induced angiogenesis; ↓ VEGF, CD31, CD34-positive vessels; ↓ IGF-induced hemoglobin levels and angiogenesis; suppression of tumour vascularization	[54,63,64,65]
A549 xenografts; patient-derived models	EGCG + metformin, BAY11-7082, or EGCG nanoparticles	Enhanced tumour suppression; ↓ Ki-67, Nrf2, HO-1, PCNA, NF-κB signalling; ↑ apoptosis (TUNEL); improved efficacy compared with monotherapy	[18,42,43]
NNK-induced lung cancer; A/J mice	0.3% green tea extract	↓ tumour multiplicity; ↓ PD-L1-positive tumour cells, supporting immunomodulatory activity in vivo	[73]
A549 xenografts	EGCG ± cisplatin	Enhanced cisplatin efficacy; ↓ *hsa-miR-98-5p* and Ki-67; ↑ CTR1 and NEAT1; ↑ ROS; ↓ ERK1/2 and ERK1/2 phosphorylation	[32,74]
Xenografts and patient-derived tumours	EGCG derivatives, nanoparticles, PBOG	Improved tumour suppression versus free EGCG; ↓ Ki-67, *p*-EGFR, *p*-Akt, *p*-ERK, *p*-NF-κB; ↑ apoptosis (TUNEL, cleaved caspase-3)	[39,41,42,60]
A549, cisplatin-resistant A549, and AXL-high xenografts	EGCG or green tea extract	↓ CSC markers (CD133, CD44, Sox2, Nanog, Oct4, CLOCK); ↑ *hsa-miR-485-5p*, *miR-485*; ↓ *p*-AXL, ALDH1A1, SLUG; suppression of aggressive tumour phenotypes (↓ Ki-67, RXRα)	[36,66,67,68]
Diet, carcinogen-, and drug-resistant models (A549, cisplatin-resistant A549, AR, H1299, xenografts)	EGCG combined with cisplatin, metformin, gefitinib, apatinib, tolcapone, osimertinib, or curcumin	↓ tumour progression (Ki-67, CyclinB1, CyclinD1, *hsa-miR-98-5p*); improved metabolic and inflammatory profiles (↓ leptin levels, STAT1, SLC7A11, IL-4, IL-8, IL-10 ↑ IL-6, IL-12, and TNF- α); reversal of drug resistance; ↑ survival; ↓ *GAS1*, *TIMP4*, *ICAM1*, and *WISP2* methylation in EGCG treatment; ↑ cleaved PARP, caspase-3	[12,19,21,22,46,47,49,69]

**Table legend:** ↑ Increased, ↓ reduced, p-phosphorylated.

**Table 5:** Summary of EGCG effects on lung cancer: clinical outcomes and therapy-associated toxicity.

Clinical Outcomes and Therapy-Associated Toxicity			
Model	Treatment	Findings	References
Stage III NSCLC (including stage IIIA/IIIB; surgically unresectable) or limited stage SCLC	Oral EGCG administered during thoracic radiotherapy ± chemoradiation (three times daily; dose range 40–440 μmol/L or 400 μmol/L)	EGCG was well tolerated with no dose-limiting toxicity; ↓ pain severity; ↓ incidence and severity of acute radiation-induced esophagitis (grade 0–1 patients); ↓ dysphagia; prophylactic use slightly more effective than therapeutic use	[96,97,98]

**Table legend:** ↓ reduced.

## Data Availability

Not applicable.
